# Lowering Barriers to Augmented Reality in Chemistry—Easy Creation of Virtual 3D Molecular Models

**DOI:** 10.1021/acs.jchemed.5c00278

**Published:** 2025-12-30

**Authors:** Frieder Loch, Johannes Huwer, Lars-Jochen Thoms

**Affiliations:** † Eastern Switzerland University of Applied Sciences, Rapperswil 8640, Switzerland; ‡ Thurgau University of Teacher Education, Kreuzlingen 8280, Switzerland; ¶ 26567 University of Konstanz, Konstanz 78464, Germany

**Keywords:** Augmented Reality, Molecular Models, SMILES Strings, Technology Acceptance Model

## Abstract

The integration of Augmented Reality (AR) into chemistry education holds significant promise but is often hindered by the effort associated with creating accurate digital molecular models. We propose two open-source tools that automate the creation of digital molecular models from SMILES strings: an online 3D molecular model generator and a Blender plugin. Two evaluations based on the Technology Acceptance Model (TAM) were carried out. The first qualitative study (*n* = 11) identified the online generator as intuitive and efficient, while the Blender plugin provided greater flexibility at the cost of higher complexity. The second study (*n* = 67) reports a quantitative questionnaire based on the TAM and showed high ratings for the online generator. Future developments should particularly address supporting users in working with SMILES notation—e.g., by integrating access to chemical databases, offering graphical molecule builders, or providing explanatory tutorials—while expanding export options toward cross-platform formats (e.g., OBJ, STL), alongside optimizing the Blender plugin’s usability to foster broader classroom adoption.

## Graphical abstract

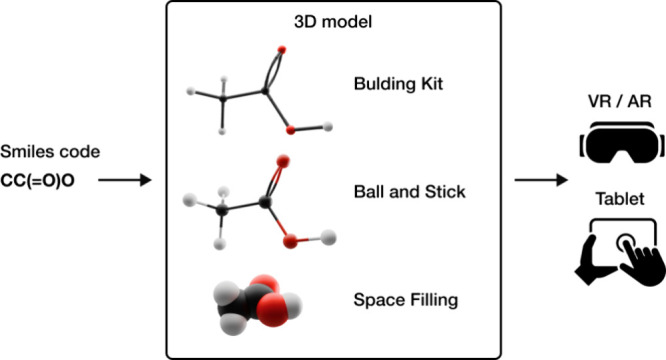

## Introduction

Molecular model kits have been an essential component of chemistry education for decades, providing students with tactile, three-dimensional tools to explore and understand molecular structures and spatial arrangements.^1^ These physical models are indispensable for grasping fundamental concepts such as molecular geometry, bonding, and stereochemistry. Their use is so entrenched in educational practices that many schools require students to purchase their own sets, underscoring their value in fostering a deeper understanding of chemistry. However, many students find it difficult to use their reasoning skills when dealing with abstract representations of scientific concepts whose understanding requires a high degree of spatial visualization.^2^ While experts can translate fluently between multiple representations,^3–5^ as well as between macroscopic, symbolic and submicroscopic levels,^6^ novices must first build up the mental models required for this.^3,7,8^ This is a process that must be scaffolded from the outside,^7,9^ for instance by using multiple representations^7,9,10^ and supplantation.^11–13^

### Augmented Reality in Chemistry Education

The advent of computational chemistry has enabled the creation of interactive 3D representations of molecules, such as ball-and-stick or space-filling models. Such representations can visualize structures that are difficult to convey through static two-dimensional figures. Moreover, augmented reality (AR) applications allow students to interact with these models to better understand molecular properties and relationships.

A long-explored application of AR is education.^14^ Literature reviews indicate the benefits of AR applications for chemistry education, especially on smartphones and tablets.^15,16^ AR has positive influences on learning,^2,17–26^ especially for visualizing and understanding abstract chemical concepts^2,26–28^ in three-dimensional representations^2,18,22,28–32^ and superimposing auxiliary information.^26,33–35^ It can provide an engaging and motivating learning environment^19,26,33–36^ and positive effects of using AR apps on attitudes toward science education,^2,37^ laboratory skills,^37–39^ and academic achievements^2,40^ have been shown. AR has been successfully used to promote explorative^31^ and collaborative^41^ learning. MoleculAR^42^ enhances textbook content by allowing students to visualize and manipulate molecular structures in real time, thereby supporting spatial reasoning and conceptual understanding of molecular geometry and bonding. MolAR^43^ scans handwritten formulas and augments them with according molecular models. The combination of tangible physical and virtual models has shown positive effects on the efficacy of chemistry education.^44^ For example, in learning chirality, students can build a physical molecular model using a virtual molecular model kit and overlay the virtual representation on the physical model to compare their model with the correct solution.^45^

AR development toolkits, such as the Apple Reality Composer Pro^46^ for MacOS and the Apple Reality Composer^47^ for iPadOS and iOS, support the creation of AR applications without programming expertise. This enables students and teachers to create their own AR applications, giving them hands-on experience with the technology. The benefits of such applications are discussed in the literature.^45,48,49^

### Creation of 3D Molecular Models

The necessary expertise to design digital 3D molecular models and their integration into AR applications that can be used in the classroom impedes the adoption of these technologies.^16,50–52^ Teachers and students often lack the expertise for designing 3D models. Therefore, AR applications often built on existing databases of 3D molecular models^49,53^ or use a combination of existing tools to create 3D models of molecules.^53^

The benefits from the availability of 3D molecular models extend beyond AR applications. 3D printing also depends on the availability of 3D models^54,55^ and provides a flexible and cost-effective method for producing tangible models that support tactile exploration.^56^ This is promising for students with visual impairments, especially for content that requires spatial understanding such as chemistry.^57^

## Requirements for the Creation of 3D Models

AR applications use 3D molecular models in different representations. Several databases of chemical molecules exist.^49,53^ However, these databases may not contain the required molecules in the correct representation, introduce additional complexity in searching, or require further processing and format conversion.

A tool for the creation of 3D models should satisfy the following requirements to be applicable in educational applications:

- **License.** The tool should be under a free and open-source license to eliminate license fees and allow its adaptation by the educators.
- **Web-based application.** This allows use in classrooms and on student devices without the need for installation or updates.
- **Intuitive interface.** An intuitive graphical user interface that includes a molecular preview lowers the entry barrier and reduces the need for training.
- **Building kit models.** The tool should create building kit models and allow for the batch creation of multiple consistent models.
- **Compatible formats.** The tool should export AR-capable formats. Especially USDZ is natively supported across Apple AR toolchains and a widely supported format for AR-based applications.^58^ The created files can be used in external applications, such as rendering engines.

## Tools for the Creation of 3D Models

Several commercial and free tools for generating 3D models of chemical structures are available^59^ and are summarized in the Supporting Information.

Previous research has proposed a workflow for the creation of molecular models using a combination of existing tools.^53^ However, this approach still requires multiple tools and format conversions. Moreover, the lack of a user interface and the possibility for batch-processing limit application scenarios in education. Other work shares similar limitations with respect to our requirements^48,60^ or focuses on different application domains.^61^

The limitations of the existing tools (e.g., license issues, incompatible formats, complexity of handling multiple tools) indicate the need for a dedicated, open, and user-friendly tool for the creation of 3D models from SMILES strings. These tools should be free and open-source and address the requirements that we outlined above.

## Contribution of This Paper

This paper presents the design, development, and evaluation of (a) an online 3D model generator and (b) a Blender plugin that supports the creation of 3D models for further processing within Blender. Both approaches support the creation of 3D models by providing an integrated workflow based on SMILES strings. Compared to existing tools, this workflow requires no prior experience with modeling software.

We evaluated the tools through two usability studies with current and prospective teachers. The first study involved 11 participants. The second study involved 67 participants. The studies addressed the following evaluation questions:

- How can the presented tools lower the entry barrier for the creation of 3D molecular models for educational purposes for people without modeling experience?
- How can the presented tools support pedagogical scenarios in chemistry education, especially with AR-based applications?
- How do users rate the tools in terms of usability and based on the technology acceptance model (TAM)?

## The OrChemSTAR Project

The tools were developed as part of the OrChemSTAR project (Organic Chemistry Science Teaching and Learning with Augmented Reality). OrChemSTAR aims to support students in learning to read and draw chemical structural formulas, as well as in translating between three-dimensional representations of chemical structures.^62,63^ The project developed a mobile application for iOS with two main functions: a learning mode and an AR mode. The learning mode prompts students to draw structural formulas, which they capture using the camera of the device. These images are analyzed to detect and visually indicate errors.^64^ The app offers an adaptive learning path based on the answers of the user. The AR mode enhances instructional materials, such as worksheets, by overlaying three-dimensional molecular models. The evaluation of the application is currently in progress.^65^ Further information on the project and the app is available on the project Web site.^66^

## Design and Implementation of a Web Application and Blender Plugin for 3D Molecular Models

This section presents the development of a web application and a Blender plugin designed for the creation of 3D molecular models. Technical implementation details are provided in the Supporting Information. Both tools use SMILES strings as the input format for molecular structure generation.

### Usage of the SMILES Notation

SMILES notation was chosen as the input format for both applications because it is internationally understood. The SMILES notation for a molecule can be searched using Wikipedia,^67^ PubChem^68^ or other chemical catalogs. In addition, many tools for drawing structural formulas offer direct SMILES export, e.g., PubChem Sketcher (also at PubChem^68^ under “Draw Structure”). By requiring the students to work with chemical databases, students acquire cheminformatics skills.

### Web Application to Create 3D Models

A web application can be accessed from any device with a browser, regardless of the operating system or the device type, and requires no manual installation. Users always interact with the latest version without depending on a third-party app store for updates. The web application is available online.^69^ The application was entirely developed by the authors of this paper and the source code is available under a permissive open-source license.^70^

Figure 1 shows the user interface and explains how to use the web application. Figure 2 shows a comparison of different representations of cyclohexane generated with the web application.

**Figure 1:**
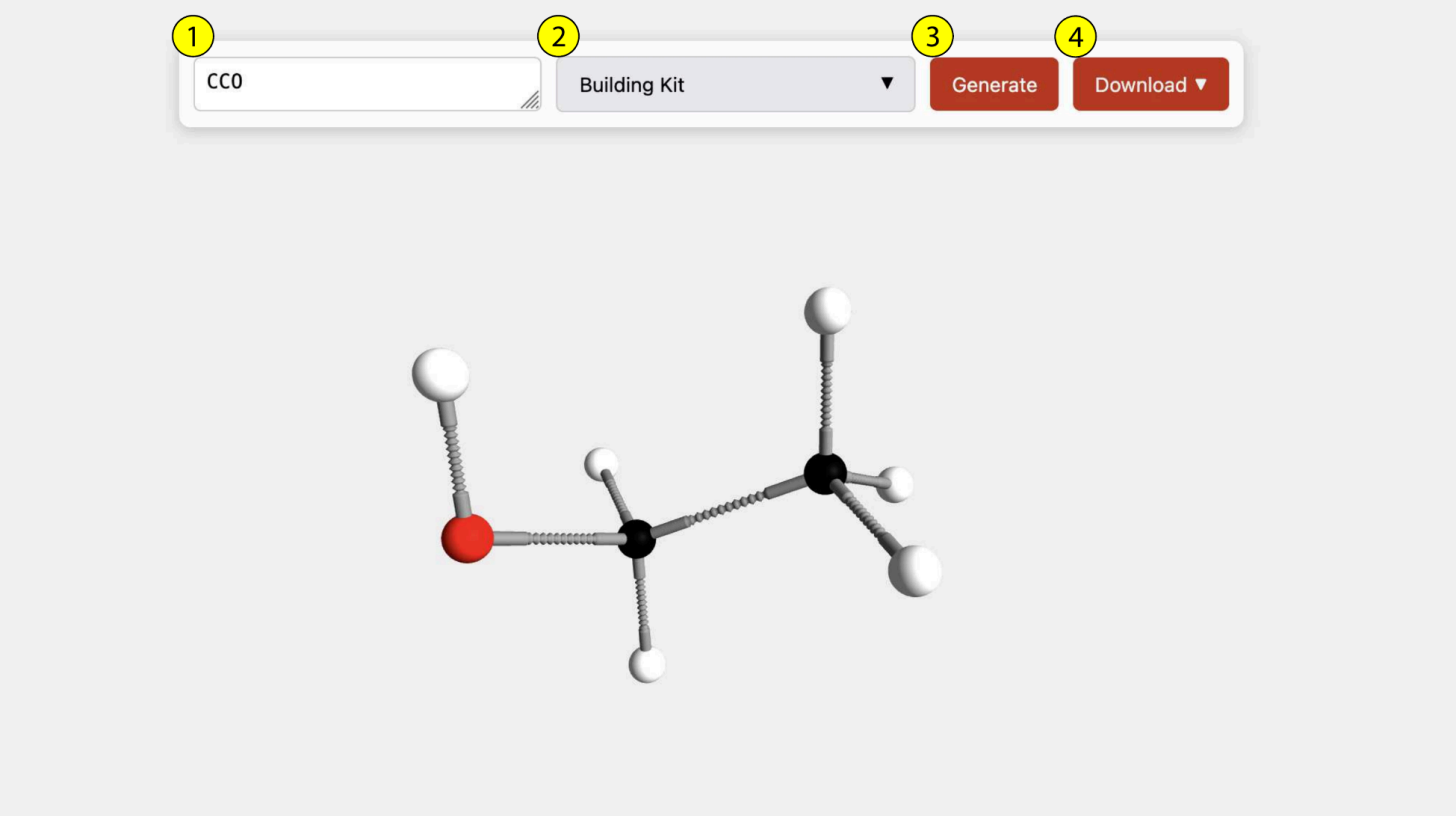
User interface of the web application. The SMILES notation of the requested molecule is entered in the input text field (1). In addition to the default building kit representation, a ball-and-stick or space-filling model can be selected in the drop-down menu (2). The “Generate” button (3) triggers the creation of a building kit representation. The representation can be rotated and zoomed with the mouse to create views from different perspectives. The “Download” menu (4) offers several options to download the molecule as an image (PNG) or in various 3D formats (USDZ, glTF, zip package of multiple USDZ files).

**Figure 2:**
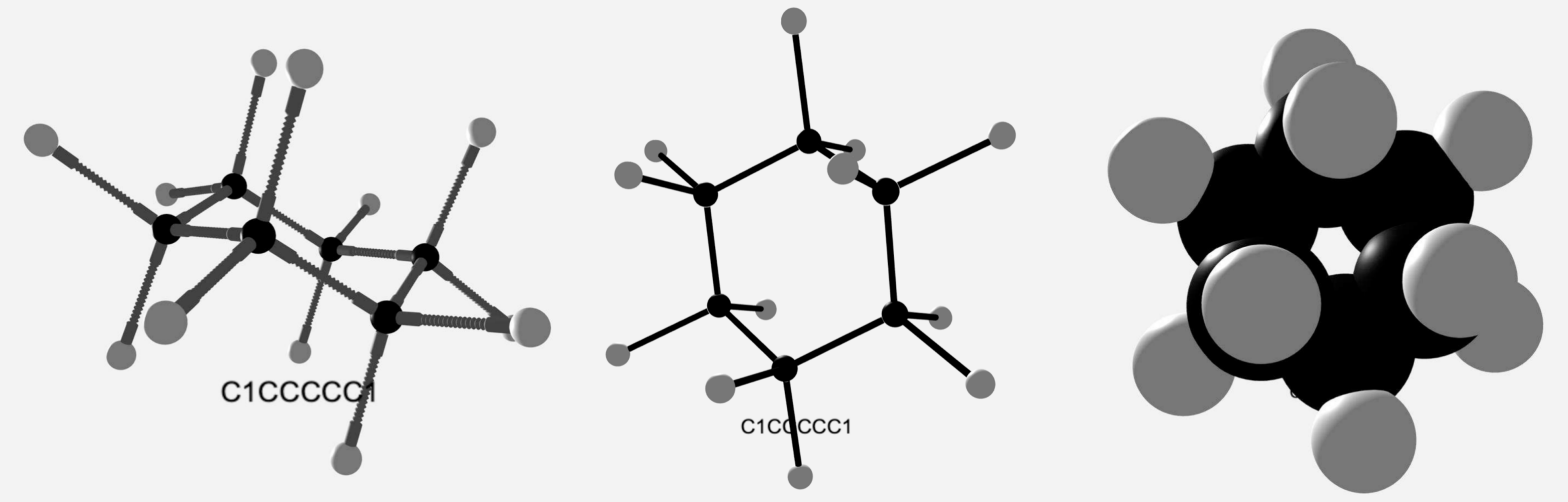
Building kit, ball-and-stick and space-filling representations of cyclohexane generated using the SMILES notation “C1CCCCC1” in the web generator.

Multiple molecular models can be generated at once by entering the SMILES notations as a comma-separated list (e.g., “CCO, CO”). The application can process up to approximately 100 molecular models in one batch. The requested molecule models are then placed next to each other in the scene and labeled with the respective SMILES notation (see Figure 3).

**Figure 3:**
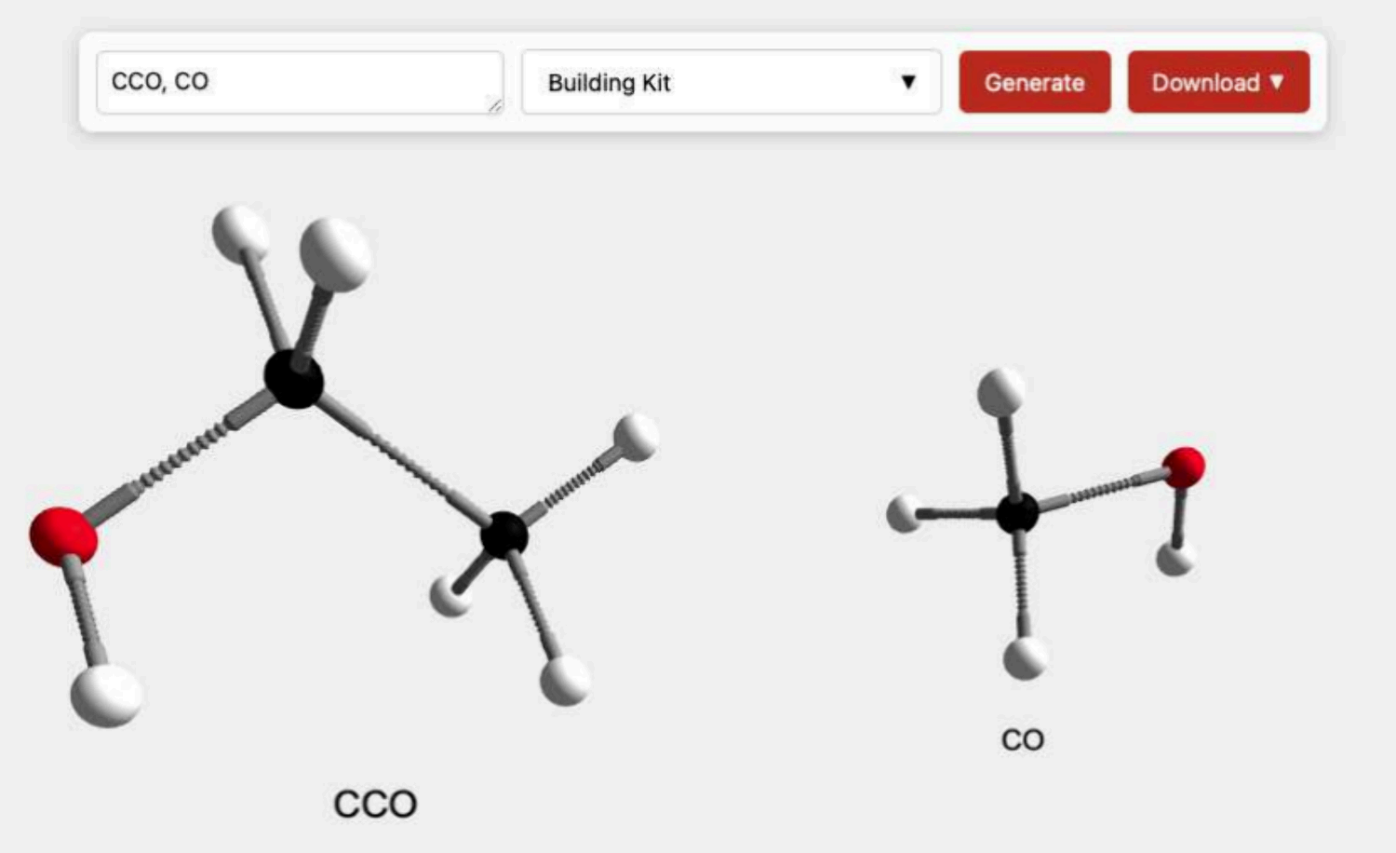
Building kit representation of ethanol and methanol generated side-by-side using a comma-separated list of the respective SMILES notations “CCO, CO” in the web generator.

The SMILES notation only contains information about the linkage of atoms within a molecule and the bonds that occur, but no information about the actual geometry (such as the actual bond angles, as would be possible with the more complex InChI notation). This means that in the case of cyclohexane derivatives, for example, no distinction can be made as to whether they are present in the armchair or tub configuration. Accordingly, all possible conformations are generated randomly, so that the tool generates a different orientation each time the user clicks the “Generate” button. This is particularly important for learners, who can thus recognize that there are several possible conformations, especially in the case of freely rotatable single bonds in hydrocarbons, where the preferred representation in structural formulas in the form of elongated chains does not reflect reality. This can serve as a starting point for a discussion of the energetically preferred spatial orientation and the real deviation from it caused by thermal energy. In this way, learners recognize the advantages and disadvantages of the SMILES notation for describing molecular geometries.

The web application provides an intuitive process, especially if the models need no additional processing. It prioritizes efficiency, enabling users to generate molecular models quickly and export them in formats compatible with AR applications. The option to input trivial names instead of SMILES strings lowers the entry barrier. Creating the model with a modeling software such as Blender offers greater flexibility for postprocessing, for instance by modifying colors or dimensions of the atoms. The following section presents a Blender plugin for the creation of 3D models.

### Blender Plugin for Creating 3D Models

Blender is a widely used modeling software. It is free, open-source, and can be extended by plugins. Educational plugins already exist and, for instance, facilitate the 3D printing of ball-and-stick models^71^ or the printing of biochemical models.^72^ Our plugin is displayed in the sidebar of Blender (see Figure 4). The plugin was developed by the authors of this paper and is available under a permissive open-source license.^73^

**Figure 4:**
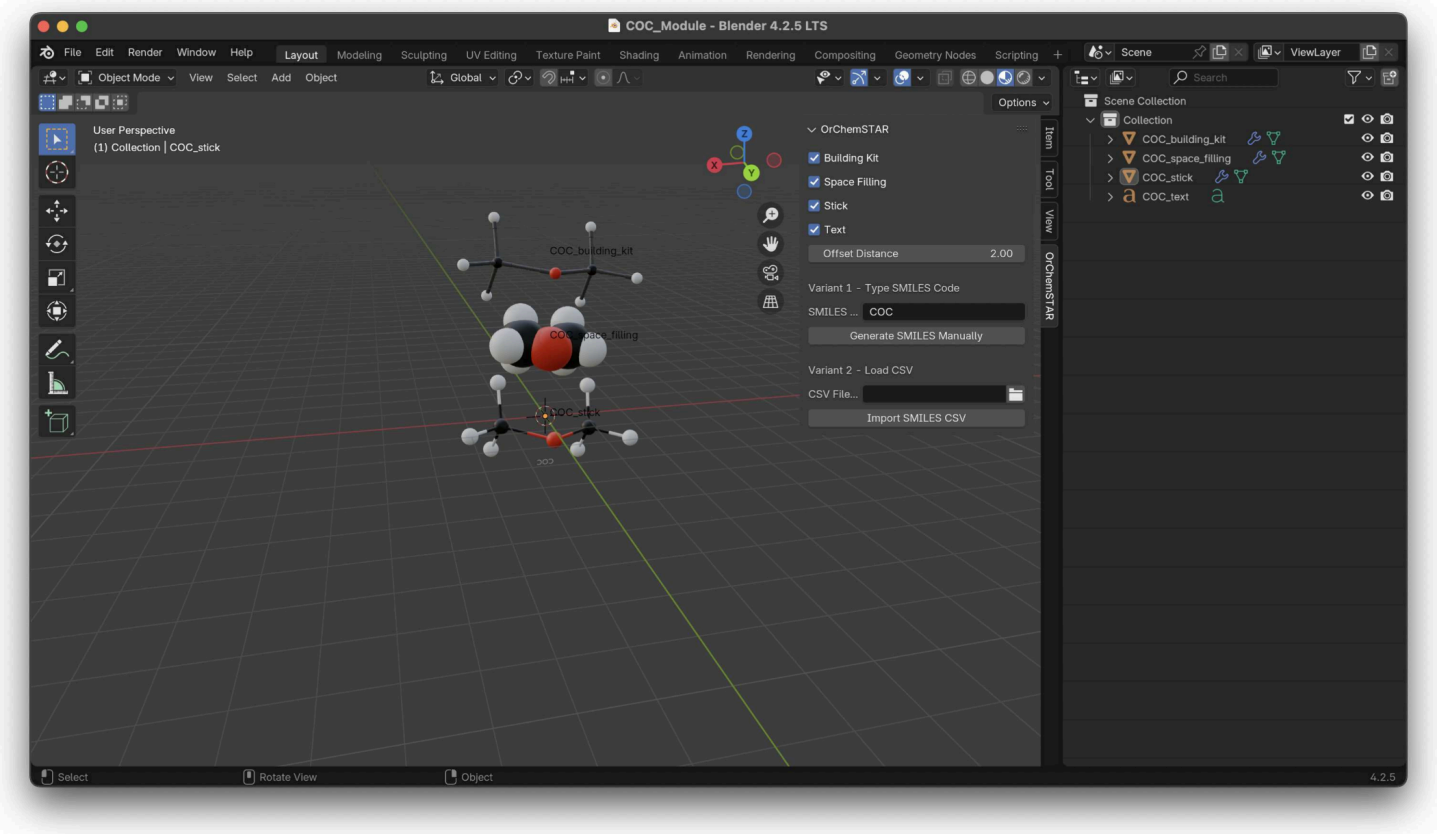
Generation of ball-and-stick, space-filling, and building-kit representations of dimethyl ether using the Blender plugin and entering the SMILES string “COC”.

The plugin accepts a single SMILES string or a list of SMILES strings in a CSV file. It can create about 300 molecular models in one batch. Thereby, educators can, for instance, create all molecules for a lecture in a consistent visual style. The user can choose among different representations (i.e., building kit, space-filling, ball-and-stick).

The Blender plugin addresses users who need more control over model visualization. It builds on the abilities of Blender and allows for precise modification of individual molecular components, offering a level of customization that exceeds the abilities of the online 3D model generator. It supports the parallel display of multiple representations, allowing for comparative analysis of molecular structures.

## Evaluation of the Tools

Both tools were evaluated with lecturers, current and prospective chemistry teachers, and students. The tests should address usability and provide feedback for further development of the tools. Both studies are based on the Technology Acceptance Model (TAM). The tests focused on the experience with using the tools and did not assess the didactic quality of the models.

We report two studies. The first study provides a qualitative comparison between both tools to identify suggestions for improvements. The second study focuses on the online generator and uses a quantitative approach with a larger sample size.

### Technology Acceptance Model (TAM)

The evaluation assessed both tools using a questionnaire based on the TAM.^74–77^ The TAM explains how users accept and adopt a technology and postulates that the intention to use a new technology is driven by two variables: perceived usefulness and perceived ease of use. Perceived usefulness is the prospective user’s belief that using an application will increase his or her job performance. Perceived ease of use refers to the degree to which the user believes that using the target system will be easy.

We adopted TAM, and not one of its extensions (e.g., TAM2,^78^ TAM3 ^79^), to focus on the individual perceptions of educators without incorporating social or organizational factors for adoption. This allowed us to evaluate the concrete appeal of the tool to current and future educators. Future work that focuses on the long-term adoption of the presented tools in educational organizations should consider additional factors present in the extended models.

### Comparison of Both Tools

#### Evaluation Procedure

Participants were instructed to interact with both tools as if they had encountered them through a pedagogic publication. The evaluation followed a three-step procedure:

1. Participants accessed the OrChemSTAR Web site and explored its content. They were asked to try the Online 3D Model Generator. We explained that the SMILES notation can be obtained from databases such as PubChem.^68^ The participants completed a questionnaire based on TAM after working with the generator.
2. We asked the participants to access the GitLab repository of the Blender plugin and install it according to the instructions.
3. After interacting with both tools, the participants completed the questionnaire again with additional items for the comparison of both tools.

The questionnaires are attached in the Supporting Information. The answers were collected and structured based on the components of TAM.

#### Participants

A total of 11 individuals participated in the study, consisting of 7 females and 4 males. The mean age of the participants was 29.6 years (SD = 7.4).

Participants frequently reported multiple professional or academic roles rather than a single designation. The sample included one high school student and one university student, alongside a larger group of early career researchers. Six participants identified as PhD students, most of whom were simultaneously involved in teaching chemistry teacher preparation courses in the Master of Education program, which trains prospective high school chemistry teachers. Two PhD students and one postdoctoral researcher combined their academic roles with high school teaching. Notably, three participants held a triple role, working as PhD students or postdoctoral researchers while teaching both chemistry education in the Master’s program and chemistry at the secondary school level. One participant was solely active as a teacher without a current PhD or postdoctoral affiliation. Overall, the sample reflects a heterogeneous group spanning the educational pipeline from pupils to postdoctoral researchers, with strong representation of individuals at the intersection of research and teaching.

All participants took part voluntarily and provided informed consent electronically before participating. Participation was anonymous, and no identifiable personal data were collected. According to Article 2 of the Swiss Human Research Act (HRA), the study does not qualify as human research, as it did not involve health-related data or medical interventions. In accordance with institutional and national ethical guidelines, no formal approval from an ethics committee was required.

#### Results for Online 3D Model Generator

Users highlighted the intuitive interface and the efficient creation of molecular models. Support for trivial names, various molecular representations, and direct AR viewing on the iPad were highlighted. However, the absence of guidance on SMILES notation poses a major barrier.

##### Ease of Use

Most participants found the operation simple and intuitive. The ability to create molecules quickly was positively emphasized. Some users noted that the SMILES notation was not self-explanatory and that a brief guide would be helpful. Novice users may benefit from an explanation on handling the export format (USDZ). Overall, the application was perceived as self-explanatory, with the only obstacle being the SMILES notation.

##### Perceived Usefulness

Participants found the application useful, especially because of the time savings compared to more complex modeling programs. The ability to quickly download molecules and view them directly in AR was appreciated. The application was seen as a valuable addition or even replacement for physical molecular construction kits. Especially for people with difficulties in the mental rotation of molecules, the 3D representation was helpful. One criticism was that a download in OBJ or FBX would be desirable.

##### Attitudes Toward Using the Online 3D Model Generator

The participants have a positive attitude toward using the application. The potential time savings and the ease of use enables quick integration into lessons. Many would use the application specifically for preparation or in organic chemistry lessons, as it is easy to use and allows students to be guided effectively.

##### Behavioral Intention to Use the Online 3D Model Generator

Many participants see potential for using the application in their studies in chemistry didactics. The potential time saving when creating 3D models for AR applications was particularly emphasized. Some would like to share the application with prospective teachers, as it provides an easy introduction to AR design. Future chemistry teachers find it useful, both for themselves and for their students, although potential hurdles need to be considered.

#### Results of Blender Plugin

Participants highlighted the ability to view multiple molecules in different representations. The concurrent display of all three labeled formats and the option to merge them into one file was valued. Although a help video was provided, users without prior experience still found Blender to be complex.

##### Ease of Use

Opinions on the ease of use were mixed. While the generation of molecules is quick and uncomplicated after installation, familiarization with Blender was described as hard. Operation was particularly challenging for users without previous knowledge, while experienced Blender users had fewer difficulties. Compared to the web-based alternative, Blender was perceived as more demanding. Another shortcoming was the inability to enter molecules using trivial names.

##### Perceived Usefulness

The Blender plugin was not perceived as an added value compared to the online generator. However, the application offers great individualization possibilities, especially for people with Blender knowledge. The greatest added value arises when it is not just a question of displaying molecules, but also of further processing options.

For school lessons, it was noted that the technical skills required by many teachers could make practical implementation more difficult. Some found the installation and operation too complex, which reduces the usefulness for simple visualization purposes.

##### Attitudes Toward Using the Blender Plugin

The participants expressed mixed attitudes. While some found it useful, others found it too time-consuming to use regularly. However, some participants saw potential for the creation of teaching materials. Nevertheless, there was interest in the application, especially for advanced users who are familiar with Blender.

##### Behavioral Intention to Use the Blender Plugin

Most participants perceived little added value compared to the online generator. However, some could imagine using it for the preparation of teaching material, especially if special molecular models are required. The application is seen as impractical for schools, while it could be useful for university teaching or research. Overall, there is interest in the application, but the high entry barrier remains.

#### Discussion

The study identified trade-offs between the online 3D model generator and the Blender plugin. Participants found the online generator easier and more intuitive due to its web-based nature and simple interface. This makes it more suitable for general educational use, particularly in secondary and undergraduate courses. Its ease of use makes it a valuable resource for teachers looking to enhance student engagement through AR.

The Blender plugin offers superior customization and flexibility, which is valuable in specific use cases. Chemistry education researchers and instructors in university settings may use the Blender plugin for creating customizable models or advanced instructional materials. However, the benefits did not justify the steeper learning curve for most users.

### Evaluation of the Molecule Generator

To further assess the applications of the online generator, we conducted an additional evaluation with a larger group. The evaluation used an online questionnaire to facilitate the distribution to a broad audience.

#### Study Design

The evaluation directed participants to examine the functions of the online generator, especially the ability to generate individual and multiple molecular models from SMILES strings. After testing the molecule generator thoroughly, the participants were directed to a questionnaire.

The questionnaire was based on the TAM, especially perceived ease of use and perceived usefulness (see Supporting Information). We focused on these aspects instead of the participants’ attitude toward using it, since we evaluate a new technology that has not yet been used in a work context. The questions were answered on 10-point Likert scales. The participants were also invited to provide additional comments as text at the end of the questionnaire.

Statistical analyses were performed using *R* statistical software.^80^ Path models were estimated with the *sem* function of the *lavaan* package,^81^ applying maximum likelihood (ML) estimation with the NLMINB optimizer. Model fit was evaluated using common indices (χ^2^/*df*, *RMSEA*, *SRMR*, *CFI*, *TLI*), without relying on strict cutoff criteria.^82^

#### Participants

A total of 67 participants took part in the study (see Table 1).^83^ Recruitment was carried out via social media and academic mailing lists.

**Table 1:** Sample Description by Group

Group	*n*	*n*_f_	*n*_m_	*n*_n.a._	*M*_age_	*SD*_age_	*n*_CH_	*n*_DE_	*n*_other_
Chemistry lecturers	14	6	8	0	41.2	13.5	5	8	1
PhD students	12	7	5	0	28.3	2.5	0	12	0
School teachers	14	3	9	2	43.0	8.6	8	6	0
Students	27	15	11	1	22.9	3.3	3	23	1

All participants took part voluntarily and provided informed consent electronically before participating. Participation was anonymous, and no identifiable personal data were collected. The study involved no foreseeable risks for participants. In accordance with institutional and national ethical guidelines, no formal approval from an ethics committee was required.

#### Results

An exploratory factor analysis confirmed the three theoretical constructs of perceived ease of use (PEU), perceived usefulness (PU), and intention to use (ITU). To examine whether a direct effect of PEU on ITU exists in addition to the indirect effect mediated by PU, we compared two structural models. Model A (partial mediation) included a direct path from PEU to ITU (*PU* ∼ PEU; *ITU* ∼ PU+PEU), whereas Model B (full mediation) excluded this direct path (*PU* ∼ PEU; *ITU* ∼ PU). A chi-square difference test indicated that the more complex Model A did not significantly improve model fit over Model B, Δχ^2^(1) = 2.40, *p* = 0.121. This suggests that the direct path from PEU to ITU is not necessary to explain the data, supporting a full mediation model in which the effect of PEU on ITU is entirely transmitted through PU.

A multigroup comparison tested whether the path coefficients differed significantly across groups. The constrained model, in which all regression paths were held equal across groups, did not fit significantly worse than the unconstrained model (Δχ^2^(6) = 3.21, *p* = 0.782). This indicates that the relationships specified in Model B can be assumed to be invariant across groups, supporting the use of a common structural model.

In the path model with direct mediation 70% of the variance in ITU is explained by PU (Figure 5).

**Figure 5:**
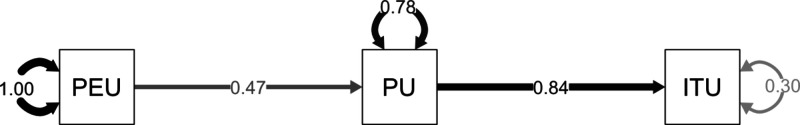
Path modeling (*n* = 42) of technology acceptance model reporting standardized coefficients (estimator: ML, optimization method: nlminb, χ^2^ = 2.4, *df* = 1, χ^2^/*df* = 2.4, *p* = 0.121, *CFI* = 0.977, *TLI* = 0.930, *RMSEA* = 0.183 [90*%* CI: 0.000, 0.493], *SRMR* = 0.047, for all coefficients *p* < 0.001).

Figure 6 shows the distribution of PEU, PU, and ITU scores by group. Across all groups, there are very high scores for PEU and high scores for PU and ITU. On all three scales, school teachers rate the Online 3D Model Generator slightly lower than the other groups.

**Figure 6:**
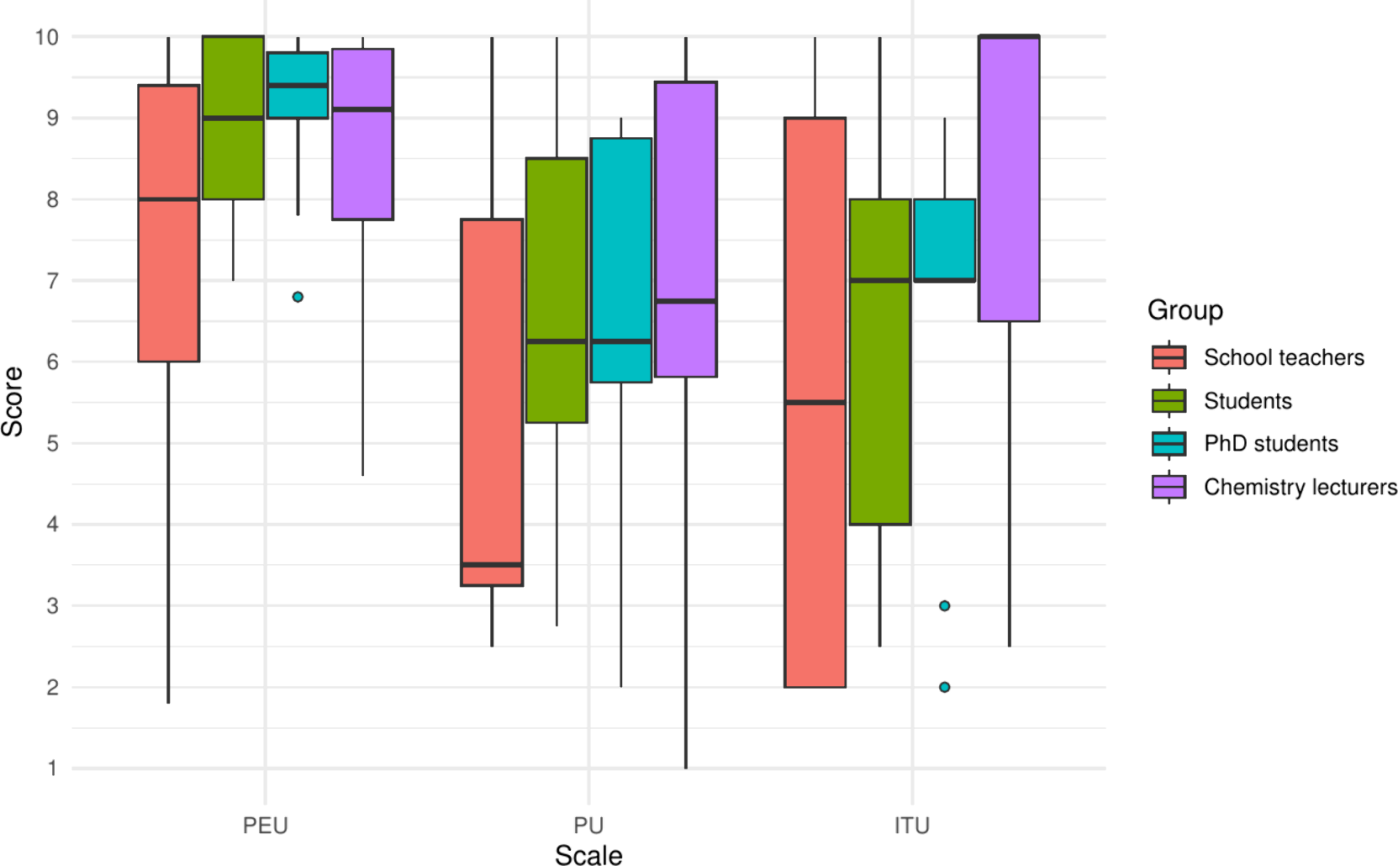
Distribution of PEU, PU, and ITU scores by group.

## Discussion

The evaluation results obtained through the questionnaire survey show that almost all respondents found the Web Molecule Generator very easy to use. The chemistry teachers surveyed rated its usefulness for chemistry lessons in schools lower than the university test groups. The comments provided by respondents clearly indicate that school teachers find the use of SMILES notation cumbersome and unfamiliar, whereas at university level, SMILES notation is considered a simple and practical reference. Chemistry lecturers particularly praised the ability to create a large number of molecules in a single call, which is useful for preparing lecture slides and exams.

## Conclusion and Outlook

The integration of AR applications into chemistry education offers substantial benefits. However, the lack of intuitive tools for the creation of 3D models hinders their broader practical adoption.

This paper introduced two tools designed to support the automatic creation of 3D models from chemical molecules. Both tools are available under the permissive MIT license to encourage community contributions and facilitate practical adoption. We assessed their potential and identified areas for improvement through two evaluations. Optimizing and expanding the functionalities of both tools based on future user feedback will be crucial in furthering the adoption of AR-based chemistry education.

The evaluations highlight the complementary nature of both tools. The online 3D model generator is a user-friendly tool for integrating AR into chemistry education. Features such as previewing molecular models and using the application without installation enhance its usability. Meanwhile, the Blender plugin caters to users requiring advanced customization of molecular representations. A workflow in Blender, for instance, allows texturing the molecules or creating animations to visualize chemical reactions.

### Limitations

This study focused on the perception of users. This was driven by the exploratory nature of the research. Therefore, we could not obtain quantitative data to address some aspects, such as potential time savings achieved through our tools. We consequently used manual coding for the open-ended responses rather than a structured qualitative analysis.

The study did not assess the presented tools in teaching scenarios since it focused on the acceptance by educators. Therefore, we cannot evaluate the didactic effectiveness of the tools in classroom.

### Possible Extensions

Several enhancements to the developed tools could be explored. These possibilities will be outlined in the following sections.

#### Supporting SMILES Strings

Currently, the tools are designed for use cases when the molecules to be modeled in 3D are already available as SMILES strings. This assumes that users possess knowledge of chemical nomenclature or have access to external databases to obtain valid SMILES strings. To make the tools more accessible for users with less knowledge, future iterations should support the creation and validation of SMILES strings. This could, for instance, be achieved by integrating a graphical molecule builder.

#### Support Blender Use

The Blender plugin needs a simpler installation process and a more intuitive interface. Providing preconfigured Blender projects could offer starting points for common workflows. Analyzing the customizations that expert users may wish to perform in Blender could guide the development of specific tutorials. Additionally, a direct USDZ export would improve the compatibility with AR toolkits.

#### Further Educational Applications

Further extensions could broaden the use cases of the online generator. The integration with Learning Management Systems (LMS) such as Moodle would facilitate the adoption in classroom settings. These extensions would allow teachers to quickly visualize various molecules during lessons and provide students with interactive exercises.

Another promising direction is the creation of gamified learning scenarios. Such scenarios could require the students to identify the name of a provided molecule or identify and correct errors in a given time frame. Such approaches could boost student motivation to improve chemistry education.

#### Accessible Chemistry Education

Students with visual impairments could benefit from the availability of tactile models. Creating tactile models of molecules requires printable 3D models, which can be challenging to generate. The creation of 3D models supports inclusive learning scenarios, enhancing accessibility for all students. The presented tools simplify model creation and make molecular structures more tangible for visually impaired students.

Several extensions to the model generation could be considered to enhance accessibility. The geometry could be optimized, for instance by enlarging critical parts. The possibility to add Braille labels to parts of the molecules could be considered to, for instance, label atoms. This would ensure that students with visual impairments can distinguish the components.

## Supplementary Material



## Notes

Implementation notes for the molecule generators, the full TAM questionnaires used in the qualitative and quantitative studies, and a comparison of existing 3D modeling tools (PDF)
