# Supplementary material for: Lowering Barriers to Augmented Reality in Chemistry—Easy Creation of Virtual 3D Molecular Models
Source: J Chem Educ. 2025 Dec 30;103(1):185–94. doi: 10.1021/acs.jchemed.5c00278 (PMC12805560; doi:10.1021/acs.jchemed.5c00278)
Supplement: Supplementary file 1 [file ed5c00278_si_001.pdf]

Supporting Information for: Lowering Barriers to Augmented Reality in Chemistry—Easy Creation of Virtual 3D Molecular Models

Frieder Loch,[†] Johannes Huwer,[‡,¶] and Lars-Jochen Thoms[*,‡,¶]

†*Eastern Switzerland University of Applied Sciences, Rapperswil, Switzerland*

‡*Thurgau University of Teacher Education, Kreuzlingen, Switzerland*

¶*University of Konstanz, Konstanz, Germany*

E-mail: lars.thoms@phtg.ch

Implementation Notes for the Molecule Generators

The OrChemSTAR Molecule Generators are based on the open-source RDKit[1] for SMILES code interpretation. The web-based generator is implemented entirely in browser-compatible web technologies and uses Three.js[2,3] for real-time 3D visualization. The Blender plugin employs the Blender Python API[4] for 3D model creation and export. Both tools are available free of charge under the permissive MIT License. This allows unrestricted use, modification, and integration into other projects, provided the original copyright notice remains intact. Comprehensive documentation and installation guides are provided in the public GitLab repositories.[5,6] Table S1 summarizes their implementation and export features.

Table S1: Implementation notes and export features of the OrChemSTAR Molecule Generators.

Tool	Implementation	Export formats / Features	Ref.
Web Generator	Browser-based tool using RDKit for SMILES interpretation and Three.js for 3D rendering.	PNG images of current projection; glTF 3D models; USDZ 3D (for AR applications, e.g., Apple RealityComposer).	[1–3,5]
Blender Plugin	Extension built with the Blender Python API to control all interface actions via Python calls.	All 3D export formats supported by Blender (e.g., OBJ, FBX, STL, glTF, USD).	[4,6]

TAM questionnaire used in the qualitative study

Table S2: Items of the open-ended TAM questionnaire used in the qualitative study.

Item	English	German
Positive comments	Please describe in as much detail as possible what you particularly liked about the last application you tested.	Bitte schreiben Sie so ausführlich wie möglich, was Ihnen besonders gut an der zuletzt getesteten Anwendung gefallen hat.
Negative comments	Please describe in as much detail as possible what you did not like about the last application you tested.	Bitte schreiben Sie so ausführlich wie möglich, was Ihnen weniger gut an der zuletzt getesteten Anwendung gefallen hat.
Perceived Ease of Use	Please describe in as much detail as possible how easy you found the last application you tested to use.	Bitte beschreiben Sie so ausführlich wie möglich, wie Sie die Einfachheit der Bedienung der zuletzt getesteten Anwendung wahrgenommen haben.
Perceived Usability	Please describe in as much detail as possible how you perceived the usefulness of the last application you tested.	Bitte beschreiben Sie so ausführlich wie möglich, wie Sie die Nützlichkeit der zuletzt getesteten Anwendung wahrgenommen haben.
Attitudes Towards Using	Please describe in as much detail as possible your attitude toward using the last application you tested.	Bitte beschreiben Sie so ausführlich wie möglich, welche Einstellung Sie gegenüber einer Nutzung der zuletzt getesteten Anwendung haben.
Behavioral Intention	Please describe in as much detail as possible whether you will use the last application you tested in your studies/profession.	Bitte beschreiben Sie so ausführlich wie möglich, ob Sie die zuletzt getesteten Anwendung in Ihrem Studium/Beruf nutzen werden.
Comparison: Better in web	Please write in as much detail as possible what you liked better about the online tool than the Blender plugin.	Bitte schreiben Sie so ausführlich wie möglich, was Ihnen an dem Online Tool besser gefallen hat als an dem Blender Plugin.
Comparison: Better in Blender	Please write in as much detail as possible what you liked better about the Blender plugin than the online tool.	Bitte schreiben Sie so ausführlich wie möglich, was Ihnen an dem Blender Plugin besser gefallen hat als an dem Online Tool.

TAM questionnaire used in the quantitative study

Table S3: Items of the TAM questionnaire used in the quantitative study.

Scale	English item text	German item text	Ref.
Perceived Usefulness	Using the OrChemSTAR Molecule Generator in my job would enable me to accomplish tasks more quickly.	Die Verwendung des OrChemSTAR Molecule Generator in meiner Arbeit würde mir ermöglichen, Aufgaben schneller zu erledigen.	7,8
Perceived Usefulness	I would find the OrChemSTAR Molecule Generator useful in my job.	Ich würde den OrChemSTAR Molecule Generator in meiner Arbeit nützlich finden.	7,8
Perceived Usefulness	Using the OrChemSTAR Molecule Generator would make it easier to do my job.	Die Verwendung des OrChemSTAR Molecule Generator würde mir die Arbeit erleichtern.	7,8
Perceived Usefulness	In my job, usage of this OrChemSTAR Molecule Generator is relevant.	In meiner Arbeit ist die Verwendung dieses OrChemSTAR Molecule Generators relevant.	8,9
Perceived Ease of Use	Interacting with the OrChemSTAR Molecule Generator does not require a lot of my mental effort.	Die Interaktion mit dem OrChemSTAR Molecule Generator erfordert keinen großen geistigen Aufwand.	8,9
Perceived Ease of Use	I find the OrChemSTAR Molecule Generator to be easy to use.	Ich finde den OrChemSTAR Molecule Generator einfach zu bedienen.	8,9
Perceived Ease of Use	I feel I have an intuitive sense on how to operate the OrChemSTAR Molecule Generator.	Ich habe ein intuitives Gespür dafür, wie der OrChemSTAR Molecule Generator zu bedienen ist.	9
Perceived Ease of Use	I have no problem with the quality of the OrChemSTAR Molecule Generator output.	Ich habe keine Probleme mit der Qualität der Ausgabe des OrChemSTAR Molecule Generators.	8,9
Perceived Ease of Use	I find it easy to get the OrChemSTAR Molecule Generator to do what I want it to do.	Ich finde es einfach, den OrChemSTAR Molecule Generator dazu zu bringen, dass er das tut, was ich möchte.	8,9
Intention to Use	If I gain access to the OrChemSTAR Molecule Generator, I predict that I will use it.	Wenn ich Zugang zum OrChemSTAR Molecule Generator bekomme, sage ich voraus, dass ich diesen nutzen werde.	8
Intention to Use	Assuming I have access to the OrChemSTAR Molecule Generator, I will use it in the job.	Angenommen, ich habe Zugang zum OrChemSTAR Molecule Generator, dann setze ich diesen in meiner Arbeit ein.	8
Dismissed	The OrChemSTAR Molecule Generator has good functionality (features).	Der OrChemSTAR Molecule Generator verfügt über gute Funktionen (Features).	9

Tools for the Creation of 3D Models

Several tools for generating 3D models of chemical structures are available.[10] Their main characteristics, strengths, and limitations are summarized in Table S4. This overview highlights why we developed the OrChemSTAR Molecule Generator specifically for educational use cases.

Table S4: Comparison of tools for generating 3D models of chemical structures.

Tool	Description	Strengths	Limitations	License / Availability	Ref.
NovoPro Labs	Web-based tool to generate 3D models from SMILES strings.	Simple browser access, no installation required.	No visual preview, limited export formats, no batch processing.	Free, online only.	11
PyMOL	Molecular visualization system with broad feature set.	Powerful, widely used in research, high-quality visualization.	Open-source version requires compilation, steep learning curve, commercial license costly.	Commercial license; open-source under BSD-like license.	12,13
ChemDraw	Software suite for 2D and 3D chemical structures.	Widely adopted in research, integrates with other tools.	Proprietary license costly, research-focused, less suited for teaching.	Proprietary.	14
Avogadro	Open-source molecular editor and visualization tool.	General-purpose molecular modeling, active community.	Missing export formats for AR/VR and 3D modeling workflows.	Free, open-source.	15
ChemDoodle	Drawing and visualization software for chemical structures.	Easy-to-use interface for structural sketches.	Limited focus on 3D models, proprietary license hinders classroom adoption.	Proprietary.	16

References

(1) Landrum, G. et al. RDKit: Open-source cheminformatics. 2025; https://www.rdkit.org.

(2) Cabello, R. Three.js: A JavaScript 3D Library. 2010; https://threejs.org/, Accessed: 2025-02-26.

(3) Ricardo Cabello and contributors Three.js: JavaScript 3D Library. 2025; https://github.com/mrdoob/three.js/releases/tag/r173, Release r173, accessed: 2025-02-26.

(4) Blender Foundation Blender Python API Documentation. 2025; Accessed on February 26, 2025.

(5) Ott, S. OrChemSTAR Molecule Model Kit Generator. 2025; https://gitlab.ost.ch/i3/web-molecule-generator, Accessed on February 26, 2025.

(6) Ott, S. OrChemSTAR Blender Addon. 2025; https://gitlab.ost.ch/i3/OrChemSTAR-BlenderAddon, Accessed on February 26, 2025.

(7) Davis, F. D. Perceived Usefulness, Perceived Ease of Use, and User Acceptance of Information Technology. *MIS Quarterly* **1989**, *13*, 319.

(8) Venkatesh, V.; Davis, F. D. A theoretical extension of the technology acceptance model: Four longitudinal field studies. *Management Science* **2000**, *46*, 186–204.

(9) Holden, H.; Rada, R. Understanding the Influence of Perceived Usability and Technology Self-Efficacy on Teachers' Technology Acceptance. *Journal of Research on Technology in Education* **2011**, *43*, 343–367.

(10) Molecular Sciences Converting SMILES to 3D model. 2025; https://molecularsciences.org/content/visualizing-molecular-structures-converting-smiles-to [illegible], Accessed on May 16, 2025.

(11) NovoPro Labs Convert SMILES to 3D structure. 2025; https://www.novoprolabs.com/tools/smiles2pdb, Accessed on May 16, 2025.

(12) Schrödinger PyMOL by Schrödinger. 2025; https://pymol.org, Accessed on May 16, 2025.

(13) Open Source Initiative The 2-Clause BSD License - Open Source Initiative. 2025; https://opensource.org/license/bsd-2-clause, Accessed on May 19, 2025.

(14) Revity Signals ChemDraw. 2025; https://revvitysignals.com/products/research/chemdraw, Accessed on May 16, 2025.

(15) OpenChemistry Avogadro. 2025; https://two.avogadro.cc/, Accessed on May 19, 2025.

(16) iChemLabs ChemDoodle — Chemical Drawing Software. 2025; https://www.chemdoodle.com/, Accessed on May 19, 2025.
